# Deep-Learning-Based Methodology for Fault Diagnosis in Electromechanical Systems

**DOI:** 10.3390/s20143949

**Published:** 2020-07-16

**Authors:** Francisco Arellano-Espitia, Miguel Delgado-Prieto, Victor Martinez-Viol, Juan Jose Saucedo-Dorantes, Roque Alfredo Osornio-Rios

**Affiliations:** 1 MCIA Department of Electronic Engineering, Technical University of Catalonia (UPC), 08034 Barcelona, Spain; miguel.delgado@upc.edu (M.D.-P.); victor.martinez.viol@upc.edu (V.M.-V.); 2 HSPdigital CA-Mecatronica Engineering Faculty, Autonomous University of Queretaro, San Juan del Rio 76806, Mexico; jsaucedo@hspdigital.org (J.J.S.-D.); raosornio@hspdigital.org (R.A.O.-R.)

**Keywords:** condition monitoring, fault detection, data-driven fault diagnosis systems, deep neural network, feature fusion

## Abstract

Fault diagnosis in manufacturing systems represents one of the most critical challenges dealing with condition-based monitoring in the recent era of smart manufacturing. In the current Industry 4.0 framework, maintenance strategies based on traditional data-driven fault diagnosis schemes require enhanced capabilities to be applied over modern production systems. In fact, the integration of multiple mechanical components, the consideration of multiple operating conditions, and the appearance of combined fault patterns due to eventual multi-fault scenarios lead to complex electromechanical systems requiring advanced monitoring strategies. In this regard, data fusion schemes supported with advanced deep learning technology represent a promising approach towards a big data paradigm using cloud-based software services. However, the deep learning models’ structure and hyper-parameters selection represent the main limitation when applied. Thus, in this paper, a novel deep-learning-based methodology for fault diagnosis in electromechanical systems is presented. The main benefits of the proposed methodology are the easiness of application and high adaptability to available data. The methodology is supported by an unsupervised stacked auto-encoders and a supervised discriminant analysis.

## 1. Introduction

The modern manufacturing environment of the industry is characterized by the integration of information systems in manufacturing processes. Such current framework, where there is an integration between production technology and information technology is called Industry 4.0 [1]. This environment is, then, defined by the implementation of multiple sensors collecting information continuously. The use of cloud-based software services allows for production data tracking, production process optimization as well as the condition assessment of machinery to prevent the appearance of critical failures. In this regard, electric motors still represent one of the key components in the manufacturing industry. Electrical Motor-Driven Systems (EMDS) are responsible for driving a large number of manufacturing processes.

The massive use of electric motors makes them the main mechanical energy supply in the industry. It is estimated that EMDS account for between 43% and 46% of all global electricity consumption [2]. In this sense, the use of induction motors and Permanent Magnet Synchronous Motors (PMSMs), stand out for being the most widely implemented. The first one is highlighted for its robust nature and low cost, while PMSMs are highlighted on account of their better efficiency and higher power density [3].

Thus, the use of PMSM is being increased in the industry [4]. In this regard, PMSMs coupled to multiple mechanical components, such as screw shafts, external bearings, couplings or multistage gearboxes, represent common industrial EMDS subjected to multiple operating conditions. In fact, due to such variability in the constitution of the systems and their working cycles, it is difficult to carry out a proper characterization of eventual faults.

In this regard, the information systems represent a key tool of many industrial processes, mainly in the condition monitoring task. Nowadays, most industrial processes collect information from different sources, making measurements of physical magnitudes such as temperatures, speed, acoustic emission, stator currents, voltages or mechanical vibrations mainly [5]. Indeed, to carry out the machine condition assessment, the non-invasive installation of sensors is one of the most accepted strategies in the industry and amongst researchers [6]. Thereby, data-driven diagnosis methods have been in high demand to identify the occurrence of faults in EMDS. Stator currents and mechanical vibrations represent the most common descriptive physical magnitudes [7,8,9]. Consequently, time domain, frequency domain and time frequency domain are approaches that have been mostly applied for the characterization of such physical magnitudes [10,11,12]. Time domain approaches are based on the calculation of a high-dimensional set of numerical features describing signal trends. On the other hand, approaches based on the frequency and/or time frequency domains, although widely applied, require an extensive knowledge of the EMDS configuration and eventual faults patterns. In fact, although a great deal of data-driven diagnosis methods have been proposed around EMDS, most of these proposals have been focused over single fault scenarios [13,14,15].

In this regard, the main challenges in the field of Condition-Based Monitoring (CBM) include the following three aspects: First, the monitoring of the signals acquired from the rotating machinery are generally different speeds, loads or both and are mixed with abundant noise; therefore, it is believed that a signal processing stage is necessary in order to help extract informative features [16]. Second, the current manufacturing environment is composing of complex systems that is equipped with several sophisticated components. Additionally, working in a different operation condition, more than one malfunction can occur simultaneously. Thus, the corresponding fault diagnosis approach should be able to detect single and multiple faults. Third, the diagnostic method must be robust enough and able to correctly characterize the different fault conditions and obtain high diagnostic precision to be reliable. To address these challenges, a multitude of fusion information strategies based on the combination of different physical quantities have been proposed. Moreover, in combination with Machine Learning (ML) techniques, represent the most promising approaches for diagnosing breakdowns related to different operating conditions in industrial machinery. In this regard, significant works, such as the one presented by Harmouche et al. [17], extract discriminatory features from the enveloping spectra of vibration signals for the diagnosis of faults in bearing balls, making use of linear transformations techniques that can be used to perform dimensionality reduction. However, this study is limited to the diagnosis of one fault in a single element of the EMDS. Moreover, Jing et al. [18] used a fault diagnosis approach based on multi-sensor data fusion to detect planetary gearbox defects under three motor speeds and load-free condition.

Hamadache et al. [19] proposed a technique for the diagnosis of bearing faults under variable speed and constant load conditions used the absolute value-based principal component analysis (PCA). Furthermore, those works that consider multi-fault scenarios under different operating conditions [20] are highly complex procedures that represent an additional difficulty of configuration and application. In recent years, Deep Learning (DL), which is a subfield of machine learning, has become a prominent diagnostic tool, redefining the latest advances in a wide range of areas, such as object recognition, medical diagnostics, image processing, recognition of voice and machine translation. In modern manufacturing systems and data-driven machine condition monitoring, DL is gaining in popularity, due to its potentiality for processing and analyzing large machinery data. Moreover, these approaches have the ability to automatically extract discriminatory information from the input data by constructing deep, with multi-layered artificial neural networks through nonlinear transformations. Recent researches have shown that the application of deep learning is capable of diagnosing faults in an effective manner.

Specifically, architectures such as autoencoder (AE), Restricted Boltzmann Machines (RBM), Convolutional Neural Networks (CNN) and Recurrent Neural Networks (RNN) have been applied [21,22,23,24,25,26]. It has been found that the application of these techniques is highly dependent on the type of available data. That is, CNN is particularly useful dealing with patterns’ extraction from images or time-frequency maps resulting from time-frequency transforms. On the other hand, AE and RNN show good results dealing with time-series-based signals. For example, Lu et al. presented a detailed empirical study of stacked denoising-AE with three hidden layers for fault diagnosis of rolling bearing components [27]. In [28], Yuan et al. compared three RNN techniques including vanilla RNN, long short-term memory (LSTM) and gated recurrent unit (GRU) techniques for fault diagnosis and prognostics of aero engine. They concluded that the advanced RNN techniques of LSTM and GRU outperformed vanilla RNN. In [29], Wang et al. introduced a 2-D CNN for gearbox fault diagnosis. The wavelet transform was performed to transfer raw sensory input into 2-D time–frequency maps and a deep convolutional neural network was used for gearbox fault diagnosis. Most studies based on DL related to the topic of research are limited to scenarios with low complexity (i.e., individual faults at specific operating condition). Just a few consider multiple fault scenarios, and even less deal with a multi-sensor data fusion and multiple-domain feature extraction methodology. In [30], Shao et al. stacked multiple RBM into a (Deep Boltzmann Machines) DBM model for fault diagnosis, whose input is frequency domain data based on Fast Fourier Transform (FFT). Sun et al. conduced a compressed sensing technique to extract low-dimensional features from raw vibration signals as input features into a Deep Neural Network (DNN) model based on stacked-auto-encoders (SAEs) [21]. In [31], time-domain and frequency-domain statistical features were extracted and fed into DBN. Then, the particle swarm optimization-support vector machine (PSO-SVM) was applied on DBN outputs for fault diagnosis. Baoguo et al. used the wavelet transform time-frequency image and convolutional network-based approach to classify motor imagery [32]. However, there is not a consensus related with the signal preprocessing stage, that is, most of the related literature deals with the consideration of multiple domains, that is, time domain, frequency domain and/or time frequency domain. Besides this, the lack of common configuration procedures leads to approaches not easily replicable to other applications. Nevertheless, these approaches currently have three main disadvantages, which is avoiding their massive application: (i) the need to have large amounts of data available, something that, in monitoring applications, cannot always be obtained; in addition to this, there is the risk of overfitting, (ii) the interpretation of the learning process is not clear, which comes to consider these approaches as black boxes, and (iii) the configuration of the multiple hyper-parameters required for each technique has not yet been clarified.

Thereby, the contribution of this work lies in the proposal of a novel data-driven monitoring methodology based on deep learning for fault diagnosis in complex electromechanical systems. To address this issue, the proposed methodology consists of two main stages. First, a deep-learning-based model that is designed with the ability to extract fault characteristic features automatically from any available domain. To achieve this learning structure, a deep learning-based technique called Stacked Auto-Encoder (SAE) or Deep Auto-Encoder (DAE) is implemented. In this sense, the present methodology offers a practical way to implement these techniques in the study of electromechanical systems. Second, a feature fusion structure that is used to integrated and discriminate in a better way the features learned in the previous stage. This stage is achieved through the use of Linear Discriminant Analysis (LDA), which aims to maximize distances between different data sets. The proposed work is organized as follows: Section 2 presents the theoretical background on the deep auto-encoder. Section 3 presents the proposed method used in this study. In Section 4, the results and validation of this proposal will be discussed. Finally, the conclusions are provided in Section 5.

## 2. Deep Learning Auto-Encoder-Based

Applications that use the Artificial Neural Network (ANN) commonly implement shallow architectures, a three-layer network traditionally being the most common, where the first layer corresponds to the inputs, the intermediate layer is a hidden layer and the final layer corresponds to the outputs.

In this regard, these classic ANNs are supplied by a set of signals obtained through feature reduction and/or feature engineering processes. This occurs since shallow ANNs have difficulty learning complex features and relationships from the raw data. Conversely, a subfield of machine learning, called deep learning, has great capabilities in the learning of features, that is, it has the ability to discover these features by itself, instead of being given, in this way, by taking advantage of the properties of deep artificial neural networks to extract complex features from the initial layers, while the final layers assign these features to the target tasks, with this process occurring automatically in an unsupervised way [33]. However, the implementation of deep networks presents a problem when put into practice; during the training process with the classic backpropagation implementation algorithm, it is not possible to update the weights through the layers, the gradient becomes too small to influence the initial layers, causing a learning deficiency. This problem is known as the vanishing gradient [34]. In recent years, different techniques have been addressed in order to solve this problem, such as the use of different network architectures or the implementation of regularization algorithms, such as the dropout method [35].

In this regard, the implementation of pre-training networks is one of the best solutions. In these schemes, each layer is trained separately, unlike training an entire architecture under one objective, and updating all network weights at the same time through backpropagation. An example of individual pre-training networks implemented in recent years are auto-encoders.

An auto-encoder (AE) is a type of symmetrical neural network that is trained in a semi-supervised manner, where the objective is to learn a new transformation of the data that is approached to reconstruct the input data. The AE learning procedure consists in two phases: encoder and decoder. Encoder takes the x vector of length k containing the set of inputs signals and transforms it to a hidden layer representation h consisting of n sparse-activated neurons via a non-linear mapping as follows:

(1) $$h=f\left(W_{e}x+b_{e}\right),$$

where f is a non-linear activation function and $W_{e}$ and $b_{e}$ are the weights and biases matrices, respectively. The commonly used activation functions include softmax, relu, tanh, sigmoid and others [36]. The sigmoid function is the most commonly used function in this process.

(2) $$f\left(z\right)=1/\left(1+e^{-z}\right),$$

**Figure 1:**
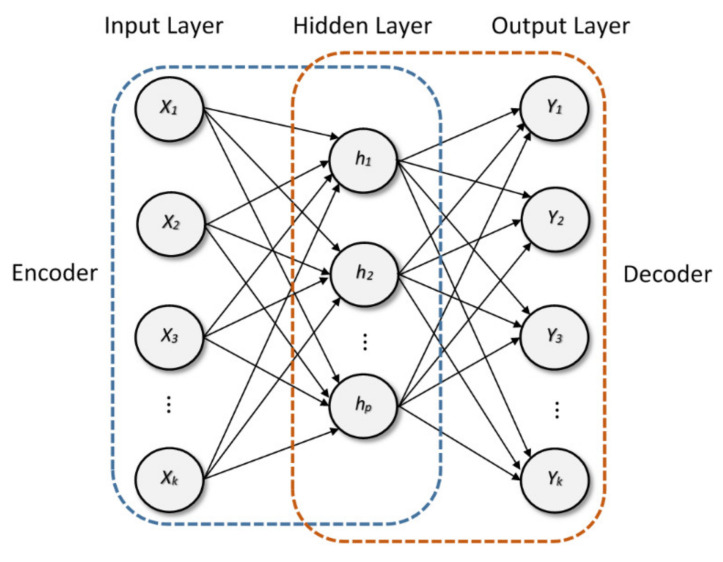
Structure of a single-layer auto-encoder with its corresponding encoder and decoder process.

Then, the encoded hidden layer was transformed to obtain the output representation of the auto-encoder through the decoder transformation in a way as follows:

(3) $$y=f\left(W_{d}h+b_{d}\right),$$

where $W_{d}$ and $b_{d}$ are the weights and biases matrices of the decoder process, respectively, and y is the output of the AE, which has the same dimension as the input x. A typical structure of a single-layer auto-encoder is show in Figure 1.

The training process of the auto-encoder consists to replicate its input at its output. This process is based on the optimization of $\theta =\left[W_{e},\;b_{e},\;W_{d},\;b_{d}\right]$ to reduce the reconstruction error between the input x and the output y by measuring of a cost function. This can be achieved by one commonly adopted measure for the average reconstruction error over a collection of N data samples (the mean squared error) and can be written as follows:

(4) $$\Omega_{Mse}=\frac{1}{N}\sum_{k=1}^{N}{\left(x_{k}-y_{k}\right)}^{2},$$

Adding a regularization term on the weights to the cost function prevents an overfitting in the network. A weight-decay regularization term is added to prevent large weights from appearing. This term is denominate the L2 regularization term and is defined by:

(5) $$\Omega_{weights}=\frac{1}{2}\sum_{i=1}^{a}\sum_{j=1}^{b}\sum_{k=1}^{c}w_{i}{\left(j,k\right)}^{2},$$

where a is the number of weight parameters, b is the number of rows and c is the number of columns in each weight matrix.

### Sparse Autoencoders

In order to generate more specialized network models that are able to discover the structure information from the input data more effectively, a sparsity regularizer is introduced. This regularizer is a function of the average output activation value of a neuron. The average output activation measure of a neuron i is defined as:

(6) $$\hat{\rho_{i}}=\frac{1}{n}\sum_{j=1}^{n}h_{i}\left(x_{j}\right)=\frac{1}{n}\sum_{j=1}^{n}f\left(w_{i}^{T}x_{j}+b_{i}\right),$$

where n is the total number of training samples. $x_{j}$ is the jth training sample, $w_{i}^{T}$ is the ith row of the weight matrix $W_{e}$, and $b_{i}$ is the ith entry of the bias vector, $b_{e}$. Therefore, a sparsity function aims to constrain the $\hat{\rho_{i}}$ values to be low, causing the auto-encoder to learn a representation, such that each neuron in the hidden layer fires to a small number of samples. This means that, each neuron functions as a specialized feature detector by responding to some feature that is only present in a small subset of the training samples. Adding a term to the cost function that restricts the values of $\hat{\rho_{i}}$ means that the neurons can become sparse. One such sparsity regularization term is the Kullback–Leibler divergence, which is a standard measure of how a probability distribution diverges from the expected distribution, where ρ is the desired sparsity parameter and $\hat{\rho_{i}}$ is the effective sparsity of a given hidden neuron. This term is defined by:

(7) $$\Omega_{sparsity}=\sum_{i=1}^{n}KL(\rho ||\hat{\rho_{i}})=\sum_{i=1}^{n}\rho \log\left(\frac{\rho }{\hat{\rho_{i}}}\right)+\left(1-\rho \right)\log\left(\frac{1-\rho }{\hat{1-\rho_{i}}}\right),$$

Finally, the cost function E, for training a sparse auto-encoder to be minimized is defined as the sum of the error term plus the regularization penalty terms, so the parameter-tuning problem can be stated as an optimization problem where the network parameters are adjusted in order to minimize the resulting cost function.

(8) $$E=\Omega_{Mse}+\lambda \times \Omega_{weights}+\beta \times \Omega_{sparsity}$$

where λ is the coefficient for the L2 regularization that controls the weight decay and β is the coefficient for the sparsity regularization term.

## 3. Fusion-Deep-Learning-Based Diagnosis Method

**Figure 2:**
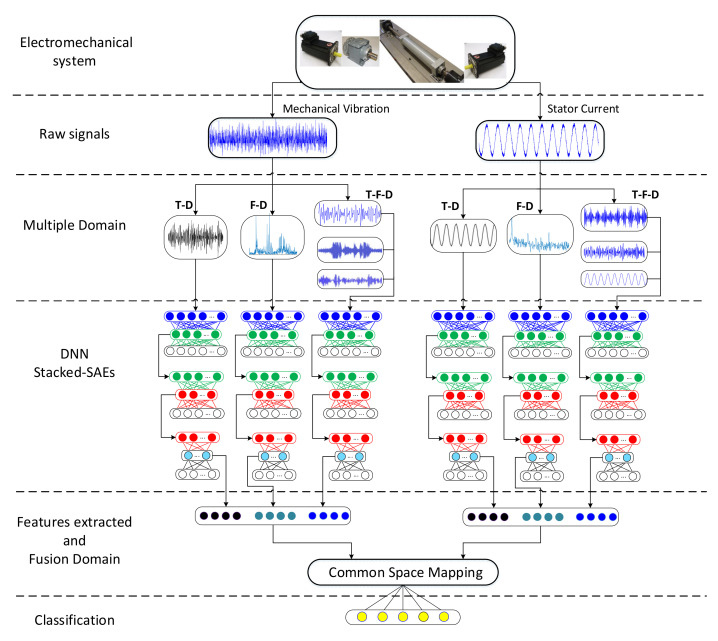
Procedure of the proposed diagnosis methodology based on hybrid feature extraction through multiple stacked-auto-encoders (SAEs) for the detection of multiple faults in electromechanical systems.

Considering the challenges posed for fault diagnosis in complex manufacturing environments, this paper proposes a step-by-step methodology that allows for the use of advanced DL tools for an information fusion scheme for fault diagnosis. In this regard, this challenge of fault diagnosis in complex manufacturing environments is addressed by implementing an SAE-based Deep Neural Network (DNN) to perform the automatic extraction of fault features through signals processed under different domains. The procedure of the proposed method is shown in Figure 2. The general structure of the methodology is intended to serve as a guide to extend such a condition monitoring procedure to multiple EMDS configurations, with their respective acquisition signals available, that intend to make use of deep learning techniques. It should be noted that the creation and implementation of modules to process data through DL tools is in turn complex, and most of the studies focus on configurations dedicated to specific EMDS, with a lack of generic methodologies that are easy to implement and adaptable to different EMDS configurations.

Studies have shown that the consideration of different physical magnitudes represent the most significant approach, aiming to reveal characteristic patterns related with different fault conditions.

In this regard, the consideration of stator current and mechanical vibration as descriptive physical magnitudes for condition monitoring represent the main source information and the most accepted approaches in the related literature [8].

Stator currents and mechanical vibration contain descriptive information close to the state of the machine. Therefore, a processing method may be required. The detailed procedure is as follows. First, the determination of the signals in three different domains: Time-Domain (T-D), Frequency-Domain (F-D) and Time-Frequency-Domain (T-F-D). The time domain consists of taking the raw signals. The classical Fast Fourier Transform (FFT) method is adopted to obtain the envelope spectrum in the frequency domain.

T-F-D processing is applied for the purpose of capturing intrinsic information from nonlinear signals. in this sense, one of the most widely used self-adaptive processing methods is Empirical Mode Decomposition (EMD). The key idea of this stage is to incorporate multiresolution into the subsequent learning process by the deep neural network. Second, by inputting the data from different domains and the different sources (vibration and current) separately, into various DNNs stacked with multiple SAEs and using the powerful feature learning ability, automatic feature extraction can be achieved. Third, the features extracted from the vibration and stator current are concatenated to summarize the information extracted from the measurement, and the Analysis Discriminant Lineal (LDA) encodes them to realize multisource fusion and is represented by means of a linear mapping of the corresponding feature space. Fourth, the representation of the extracted multiresolution features is used to generate the probability of each condition of the system by applying a simple ANN classification step.

Such proposed diagnosis methodologies provide two significant advantages: (1) the automatic learning and discriminative features through the network training process, without requiring manual feature selection, and (2) the developed multiresolution fusion technique not only improves the effectiveness of feature extraction but is also adaptive to the variability of system conditions and the presence of various faults, which occur in different components and with a different nature.

### 3.1. Signal Processing

The use of a single domain space is popular in the application of fault diagnosis in electrical machines. However, different domain spaces in the signals considered through acquisition process may have valuable and implicit information; existing methodologies that implement advanced DL techniques do not simultaneously extract information from multiple spaces to characterize the machine condition. Moreover, in industrial processes involving rotating systems, the signals contain dynamic and noisy data, which could lead to poor overall performance of machine health monitoring systems. Therefore, this paper proposes a parallel training model formed by using T-D data, F-D data and T-F-D data. The data from the different domains for mechanical vibration and stator current signals are used as inputs of different SAEs. This improves the ability to capture different characteristics in different domains and relevant information on the condition of the machine.

Then, a classical frequency analysis has been carried out on the current and vibration signals of the stator. Fast Fourier Transform (FFT) is applied to every signal sample to obtain the corresponding frequency amplitude. Several studies have focused on extracting specific fault characteristic frequencies through the envelope spectrum or using the FFT [37,38,39,40]. The results have been prominent in detecting specific faults in some components of rotating systems, i.e., bearings or gearboxes; however, for the correct application of these analyses, it is necessary to know in detail the configuration of the components that compose the system under study. In addition, the complex manufacturing environment, under the interaction of multiple components, under multiple operating conditions and the risk of multiple faults, causes these characteristic frequencies to be attenuated by noise or to overlap with other frequencies inherent to the system and of its environment, making it difficult to recognize fault patterns. Therefore, with the implementation of a DL-based technique, it is possible to extract features over signal along the time axis, which are more robust to the randomness of the motor running. Some deficiencies presented by the frequency analysis, such as abundant harmonic components, make it difficult to distinguish between the characteristic frequencies of faults, which can be overcome with some frequency band decomposition methods. The Hilbert–Huang transform (HHT) is a self-adaptive processing method for nonstationary and nonlinear signals. The Empirical Mode Decomposition (EMD) is an HHT-based signal demodulation that recursively scans local minima or maxima and then estimates the lower or upper envelopes through spline interpolation: cubic, polynomial and/or smoothing [41]. The EMD generates a frequency bands of complete and quasi orthogonal intrinsic mode functions (IMFs), which represent the intrinsic oscillation modes integrated in the signal. Thus, the faulty modes’ characteristic could be extracted through this process. In this regard, the signal decomposition in the T-F-D with the estimation of the IMFs from the vibration signals and current signals is done by the EMD. The estimation of those IMFs that best preserve the data variance from vibration signal and the current signal are fed into different DNNs stacked with multiple SAEs in the next stage for feature extraction.

### 3.2. Feature Extraction

**Figure 3:**
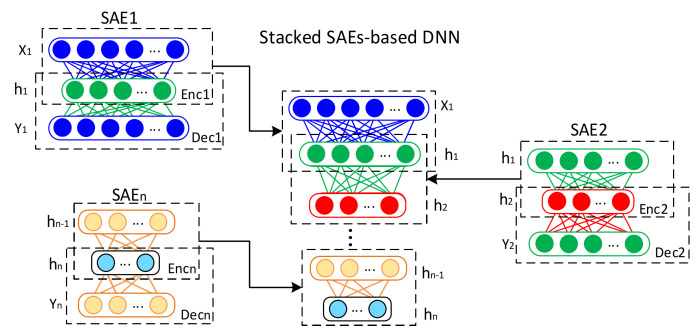
Schematic Deep Neural Network (DNN) construction and training.

Several auto-encoders are stacked to build a DNN, as shown in Figure 3. The stacked-SAEs-based DNN aims to extract intrinsic relationships and establish the mapping of complex features between the sampled signals and fault conditions of EMDS. Therefore, this architecture contains multiple hidden layers, each of which can learn a non-linear transformation from the previous layer. This layer-by-layer training method proposed by Hinton et al. [42] can be used to train a DNN. The number of input neurons is the dimension of data points considered for each domain. An unlabeled $X_{TD}$, $X_{FD}$ and $X_{i-TFD}$ training set is used for DNN layer-by-layer unsupervised training.

First, input $X_{1}$ is inputting to the first Stacked-AE, SAE1, where the first hidden layer $h_{1}$ is the coding vector calculated and the output $Y_{1}$ is the reconstruction. $X_{1}$ and $h_{1}$ are also the input and the first hidden layer, corresponding to the DNN. The hidden layer $h_{1}$ is the input of the next Stacked-AE, SAE2, which is trained to obtain the encoding vector $h_{2}$, which corresponds to the second hidden layer of the DNN. Each of the Stacked-AE trained parameters, $\theta =\left[W_{e},\;b_{e},\;W_{d},\;b_{d}\right]$, follows the learning process described in Section 2; therefore, the parameters of each encoder stage are shared to form the DNN architecture. Finally, the SAEn is trained and the DNN is initialized according to the previous steps up to the n-th hidden layer. The last output layer of DNN corresponds to the extracted multiresolution features space that will be processed to obtain the representation of extracted features.

As mentioned previously, the creation of architectures to process data through DL tools can become a complex task. The selection of hyper-parameters and the configuration of the structure is extremely related to a good reconstruction of the data, which results in having a high identification precision. The influence of the parameters in the diagnostic methodologies have been recently studied, since there is no unique or trivial configuration for the implementation of DL techniques. Most of these studies are carried out experimentally, where they obtain the specific parameters that help the implemented methods have a high diagnostic accuracy.

In this sense, and considering that we are facing an optimization problem in which the network parameters must be adjusted to minimize the cost function, a process of optimization of the key parameters for the construction of DNN is carried out. In this paper, an optimization of the hyper-parameters is performed by a genetic algorithm (GA). This optimization is carried out, individually, for each domain space considered as follows: a logical vector containing the key parameters of optimization to minimize the cost function (8) and the unit numbers of the hidden layers. The DNN parameters are as follows: the coefficient for the L2 regularization term, the coefficient for the sparsity regularization term and the parameter for sparsity proportion.

The proposed architecture consists of creating a DNN with stacked automatic encoders as shown in Figure 3, which contains an input layer, an output layer and several hidden layers, which are trained under the proposed procedure. Experimental search or random search methods are commonly used to determine hyper-parameters. However, the time-consuming search process becomes unacceptable as the range of parameters increases. In this study, some of the hyper-parameters are pre-set, and other parameters are determined through an optimization process. The number of units of the input is determined by the size of the samples of each domain. The unit numbers of the hidden layers is also obtained through the GA optimization process. The unit number of the output layer in this application is determined at 2 to generate a 2-dimensional space that allows the data to be represented in a visual way. Therefore, an initial population is randomly generated considering that at least one of the parameters contained in this initial logical vector must be selected to be evaluated; moreover, more than one element can be evaluated. In order for the optimization algorithm to converge on desired values, some constraints are imposed on each of the parameters contained in the logical vector.

Once the initial population is defined, the fitness function is evaluated, which is based on the accumulation of the data variance. With this initial population, the cost function is evaluated, which corresponds to minimizing the reconstruction error through Equation (8). Then, a new population is generated through the roulette-wheel selection process and applying a mutation in the GA, which, based on the Gaussian distribution, generates another population. Then, the process is repeated iteratively until finding the best set of hyper-parameters that perform a better reconstruction of the data. The objectives for stopping the algorithm are controlled by different aspects, such as achieving a minimum reconstruction error or reaching a maximum number of generations. Due to the high complexity of search, the criterion of maximum number of generations is more accepted.

### 3.3. Multisource Fusion

After extracting features for each domain, all the sets of features are subjected to a fusion and compression process, in one word, a base transformation by means of LDA. Through this fusion and compression process, a new set of features are represented by means of mapping, and these features are a combination of different weights from the set of extracted features by the DL process.

Therefore, the extracted features are characterized in a 2-dimensional space, allowing for a visual interpretation of the considered conditions. Additionally, this resulting 2-D common space mapping facilitates the classification task, since it works only with two input nodes.

Linear discriminant analysis is one of the most well-known supervised feature fusion and dimensionality reduction techniques in multi-class problems. The objective of LDA is to find projections in a low-dimensional representation to maximize the linear separation between the most discriminant information belonging to different classes. The criteria used for linear discriminant analysis is to evaluate the compactness within each class through the computation of the within-class scatter matrix, $S_{w}$, while computing also the separability of different classes through the scatter matrix, $S_{b}$.

The compensation between such computations lies in a new space, improving discrimination capabilities among classes. LDA aims to find a linear transformation matrix, $M\in R^{Cxd}$, mapping the original C-dimensional space into a reduced d-dimensional feature space with d < C, for which the between-class scatter matrix is maximized, whereas the within-class scatter matrix is minimized. Considering, again, M in the data set, each $m_{i}$ belongs to a class $z_{i}=\left\{1,2,\ldots ,z\right\}\;$. Let $y_{i}$ be the number of data points in the ith class and y be the number of data points in all classes. Then, the between-class scatter matrix, $S_{b}$, and the within-class scatter matrix, are as follows:

(9) $$S_{b}=\sum_{k=1}^{z}y_{k}({µ}_{k}-µ){\left({µ}_{k}-µ\right)}^{T}\;,$$

(10) $$S_{w}=\sum_{k=1}^{z}\sum_{m_{k}\in z_{k}}y_{k}(m_{1}-{µ}_{k}){\left(m_{k}-{µ}_{k}\right)}^{T}\;,$$

where ${µ}_{k}=1/y_{k}\sum_{m_{k}\in z_{k}}m_{k}$ is the mean of the data poits in the ith class, and $µ=1/y_{k}\sum_{k=1}^{z}m_{k}$ is the mean of the data points in all classes.

### 3.4. Classification

The proposed diagnostic methodology is based on the implementation of a diagnostic model for the extraction and fusion of features from multiple sources of information. Therefore, a wide characterization is obtained by applying the proposed hybrid characteristics reduction and fusion scheme to the available information set. It should be noted that the proposed hybrid feature fusion allows a simple configuration for the classification task since the input data are reduced to two dimensions. In this regard, a simple structure of an ANN-based classifier is used to obtain the diagnosis calculation of all the considered conditions. Indeed, the classifier has a classical three-layer structure. The input layer is composed of two neurons corresponding to the 2-D feature vectors resulting from the proposed hybrid feature fusion methodology. The hidden layer for this network has eight neurons following classical recommendations [43]. The output layer is composed of the number of conditions considered. This simple and classical ANN structure has been successfully implemented in different condition monitoring schemes [23].

In addition to the fault diagnosis, the proposed ANN also offers the corresponding probability value as the sigmoid function is used as an activation function in the output layer; therefore, the classification result in the ANN is related to a diagnosis probability. The training process uses the back-propagation method for calculating the gradient and the scaled conjugate gradient as a minimization technique.

## 4. Experimental Setup

In order to verify the performance of the hybrid-feature-extraction-based methodology in front of different complexity degrees, an experimental test bench based on an electrical motor-driven system has been considered. The experimental EMDS is based on two identical featured face-to-face motors, the motor under test and the motor that was applied as a load. These motors are connected by means of a screw and a gearbox, constituting the experimental setup. A motor drives the input axle of the gearbox. The output axle of the gearbox runs the screw, which, in turn, displaces the movable part. The motors are two SPMSMs with 3 pairs of poles, a rated torque of 3.6 Nm, 230 Vac, and a rated speed of 6000 rpm provided by the ABB group.

**Figure 4:**
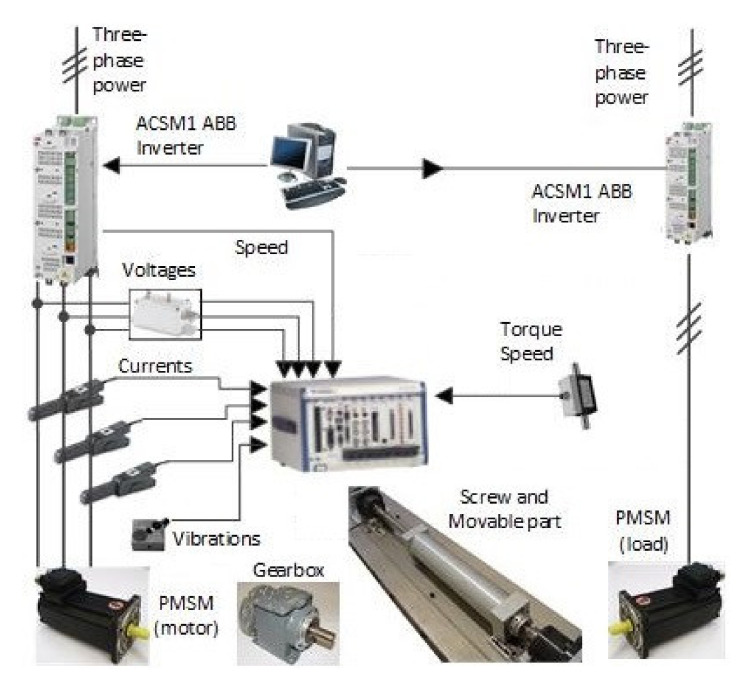
Test bench diagram used for the study of the fault diagnosis.

The motors were driven by ABB power converters (ACSM1 model). The measurement equipment is focused on the acquisition of vibrations and current signals. The accelerometer transducer and current probes, respectively, were connected to a PXIe 1062 acquisition system provided by National Instrument. The sampling frequency was fixed at 20 kS/s during 1 s for each experiment. The experimental set up diagram is shown in Figure 4.

In this study, five electromechanical actuators with identical structures but different incipient faults have been investigated. First, a complete healthy EMDS has been tested. Second, a partially demagnetized motor was developed during the fabricate with a 50% of nominal flux reduction in one pair of poles. Third, an assembly was carried out with degraded bearings. In order to cause a generalized rough defect, the non-end bearing inner as well as outer races have been scraped thoroughly. Fourth, a static eccentricity was induced through a screw attachment in the gearbox output shaft. Fifth, two gear teeth were smoothed out to impose a degradation degree on the reduction box. The test motors were supplied by two power frequencies (30 and 60 Hz) and loaded by two loading conditions (40 and 75% of the nominal load). We labeled the power supply conditions as Ps1 = {30 Hz} and Ps2 = {60 Hz}, and the load conditions as Lc1 = {40%} and Lc2 = {75%}, to refer to simple operating conditions. The labels 2Ps = {30 Hz, 60 Hz} and 2Lc = {40%, 75%} refer to a double speed or double load condition, respectively.

Therefore, up to nine combinations of operating conditions can be obtained to generate the analysis of the experiments. For each of the experimental cases, two hundred complete acquisitions were carried out. Overall, two hundred samples represent a number of valid samples to be obtained during the occurrence of a fault in an operating system. It is important to take into account that the measures considered are one second of the acquisition time. Furthermore, this number of samples is common and sufficient for the representation of healthy conditions of electromechanical systems as used by other authors [26,33,44]. In order to verify the robustness of the proposed method under variable operating conditions, the database considered during the proposed method validation has been divided in three different parts: the first one composed of 70% of the available samples for training purposes, the second one composed of 20% of the samples for validation purposes, and, finally, the third one composed of 10% of the samples for test purposes. It has to be noted that, in regard with the data set for training, a 20% in absolute terms have been used for the selection of the hyperparameters and, then, the remaining 50% has been used to train the proposed model architecture.

In addition to such data set partitioning, a five-fold cross-validation approach is applied over the data in order to corroborate that the results are statistically significant. The application of this statistical method is common in this type of approach precisely with the aim of avoiding overfitting in the structure of the model.

## 5. Results and Validation

The proposed diagnostic procedure is implemented under the Matlab programming environment, which is used to process the acquired signals and to generate the fault diagnosis model. According to the fault diagnosis schemes of electromechanical systems, the information related to the faulty conditions of the rotating machines is reflected in the appearance of perpendicular vibrations in the axis of rotation [20]. As for the stator currents, some faults cause phase modulation in the motor current and affects the magnetic field generated by multiple frequency components. Therefore, the study of this methodology will be carried out around the measurements of mechanical vibrations of the plane perpendicular to the axis of rotation of the motor, while for the study of current, one of the phases of motor supply will be taken.

**Figure 5:**
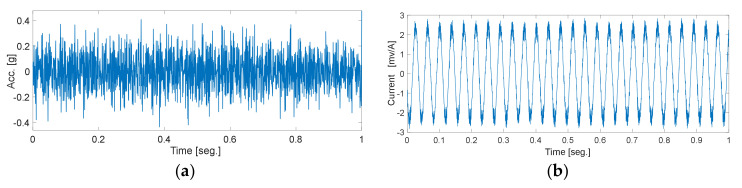
Raw data from the same time step. (**a**) Time domain raw vibration signal and (**b**) raw current signal.

**Figure 6:**
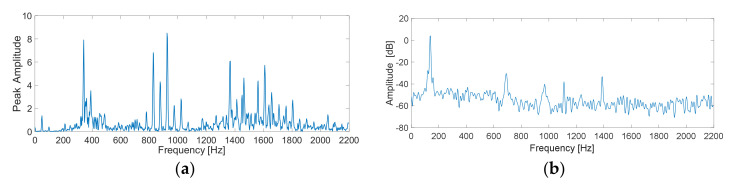
Corresponding spectra of: (**a**) vibration and (**b**) current signals.

**Figure 7:**
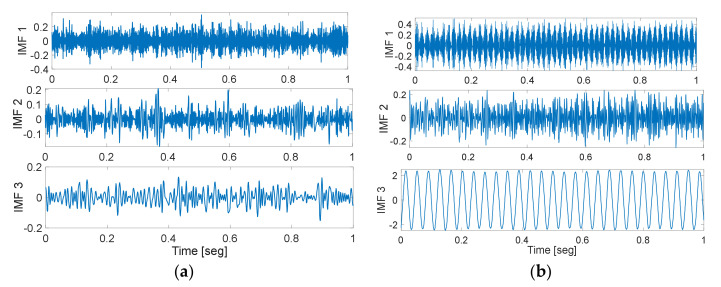
Intrinsic Mode Functions (IMFs) decomposed components using Empirical Mode Decomposition (EMD) from: (**a**) vibration signals and (**b**) current signals.

In order to characterize the condition of the machine in a more comprehensive way, parallel processing of the vibration and current signals is carried out. This way of considering the information also allows us to have a generalizable methodology in which, if the nature of the fault is unknown, that is, if it is of mechanical or electrical origin, this proposal is capable of adapting to the most descriptive source of information to characterize the different faults present. Thus, each sample is a raw vibration signal and raw current signal containing 2000 data points. Meanwhile, FFT and EMD are applied to each sample signal in order to obtain F-D and T-F-D data, and thereby generate a Multiple-Domain-Features (MDF). Therefore, the raw data, F-D data, and T-F-D data contain 2000, 1000, and 2000 data points, respectively. For the T-D, raw vibration signals and raw current signals were used, as shown in Figure 5. An example of the resulting spectra for a vibration and current signal in the frequency domain are shown in Figure 6. Three main intrinsic mode components are shown in Figure 7.

Although the MDF set contains a large part of the information related to the operating conditions of the electromechanical system, but only a few will have representative information. In this sense, the set of MDF is then analyzed through a proposed hybrid features extraction and reduction model based on deep learning to generate a space with the most discriminative features.

As described above, the process of configuring hyper-parameters and building the model based on deep learning is not trivial and requires determining a specific architecture for each application. First, the optimization process is performed by means of a GA. The main setting parameters of the GA are defined as follows: a population of five for the number of individuals, previously defined in the methodology, the maximum number of generations is established to 50 empirically, and a roulette-wheel selection scheme is fixed together with a mutation algorithm based on a selection function with a Gaussian distribution. The optimization process is applied individually to the considered MDFs. Therefore, the GA’s goal is to find the optimal hyper-parameters with which the SAEs-based model DNNs get the minimum reconstruction error. The final hyper-parameter settings for vibration and current signals are shown in Table 1 and Table 2, respectively.

Then, the space of extracted features is introduced to a stage of fusion of features through LDA, in which all the MDF are subjected to the compression procedure. Therefore, the new space of extracted features through LDA is projected in a 2-dimensional space where it is possible to achieve a visual representation of all the conditions considered. Finally, the classification task is carried out by applying a multilayer ANN-based classifier. Because the MDF space has been reduced, the input to the classifier consists of 2 units, a hidden layer and an output layer that corresponds to the number of conditions considered. The neurons in the hidden layer are set to 10 and a probabilistic sigmoid function is used as activation function is implemented. The back-propagation algorithm is used to train the classification network. The averaging diagnostic accuracy is introduced as performance metrics. The results of the classification of the proposed methodology based on the extraction of multiple domain features and information fusion for each of the 9 study cases, described in the experimental setup section, are presented in Table 3.

In order to verify the robustness of the classification process and confirm that the results are statistically significant, the classifier is trained and tested under a five-fold cross-validation scheme. Therefore, five classification indexes are obtained as a result of five iterations with complementary partitions of the original database from training and test sets. The averaged classification rates of 93% for the training and 92.03% for the test have been obtained, with a standard deviation of +/− 1.05.

The averaged classification for the test corresponds to the fusion column of the proposed approach in the Table 3.

First, the results obtained will be discussed under the proposed approach of information fusion and without fusion. In this regard, the average of the accuracy of the proposed method of 92.03%, is compared to the results from current fusion, which the average precision is 35.0%, it should be noted that current fusion results are unsatisfactory. These results are in alignment with previous research [44,45,46]; this is due to the nature of the faults, since they are mechanical. The fusion model for vibration signals presents an average accuracy of 88.6%, which shows that vibration signals are more sensitive to motor faults. However, with our proposed approach, an average of 3.36% more resolution is gained. In general, by incorporating information from multiple sources, the proposed model produces better diagnostic performance, it also has the ability to extract the best from each of the information sources.

However, it should be noted that the fusion approaches for each source of information, that is, current, vibration and current-vibration, in most situations, overcome the analysis of individual domains. In addition, for the conditions considered, vibration analysis in the frequency domain is the approach that produces the highest diagnostic precision among the domains studied. In some cases, this produces better results than the fusion approach, for example, in vibration in FD, + 3.6% more than its corresponding fusion approach. Nevertheless, having expert diagnostic knowledge and knowing with certainty which information signal and under which domain is not something that is always available. Therefore, a contribution of our proposed model is that making use of different sources of information and the analysis of different domains, it is possible to achieve high diagnostic accuracy and approximate to a maximum value of the results.

**Figure 8:**
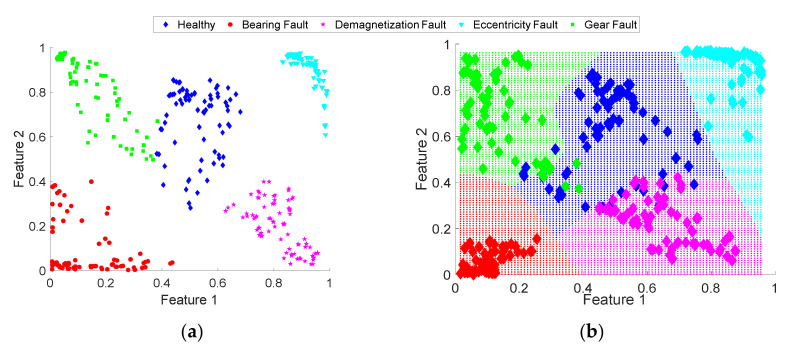
Projection of the extracted set of features resulting from proposed approach: (**a**) 2-dimensional space of each one of the conditions; (**b**) projection of the map resulting from the Artificial Neural Network (ANN)-based classification algorithm.

In order to interpret the distribution of the considered conditions, it is projected into a 2-dimensional space where it is possible to obtain a visual representation of the extracted features. Figure 8a shows the projection of the extracted set of features resulting from the application of the proposed MDF extraction and fusion approach for the operating conditions of 2Ps and Lc1. From the projection, it can be seen that the features associated with the different health conditions of the electromechanical system are generally clearly separated from each other. Only a few test samples are grouped incorrectly. Specifically, some data points of the healthy condition and gear fault overlap with each other. Moreover, a small number of data points of the healthy condition and demagnetization fault overlap. While the bearing fault condition and eccentricity fault are clearly separated. The quality of the learned essential features is shown, and we conclude that the proposed method has a high robustness to correctly identify each one of the presented fault conditions.

Finally, for the fault classification process, a multilayer ANN-based classifier with a sigmoid function was implemented, with which it is also possible to obtain the probability of belonging of each class. Through this classifier, it is possible to obtain the decision regions for each condition of the system, as shown in Figure 8b.

### 5.1. SAE Performance Discussion

**Figure 9:**
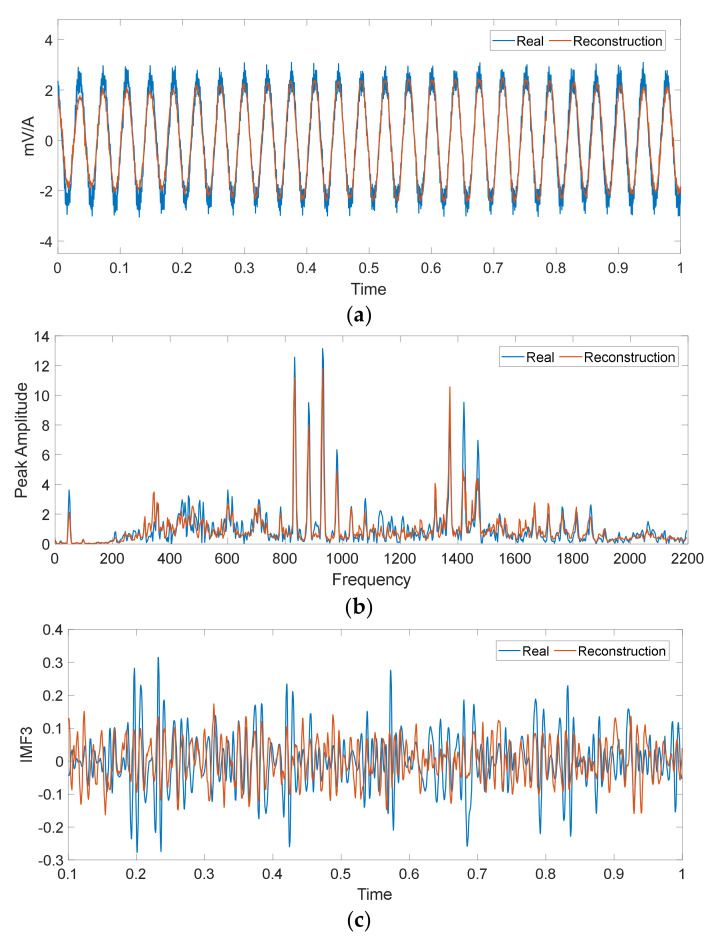
Comparison between the input signal and the reconstruction obtained using the DNN Stacked-SAEs-based feature extraction model for the healthy condition, (**a**) current in T-D, (**b**) vibration in F-D (**c**) one IMD for vibration in T-F-D.

To give an understanding of the high effectiveness of our method, the characterization of some signals obtained through the DNN Stacked-SAEs-based model are presented. As mentioned above, an optimization process was applied to find the optimal hyper-parameters through GA in order to minimize the rebuild error. The characterizations for some signals are presented in Figure 9.

The signals presented correspond to the training result of the model for the current analysis in TD for the conditions Ps1 and Lc2, considering the healthy state in Figure 9a, for the vibration analysis in F-D in Figure 9b and for the vibration analysis in T-F-D in Figure 9c. It can be seen that the signals obtained through the reconstruction closely matches the target on most of the signal, presenting a low average reconstruction error, with the mean squared error being at an average value of 0.014 during training and 0.019 during validation, for the all data set, where the most convincing value is 0.001, while the maximum error obtained was 0.026.

**Figure 10:**
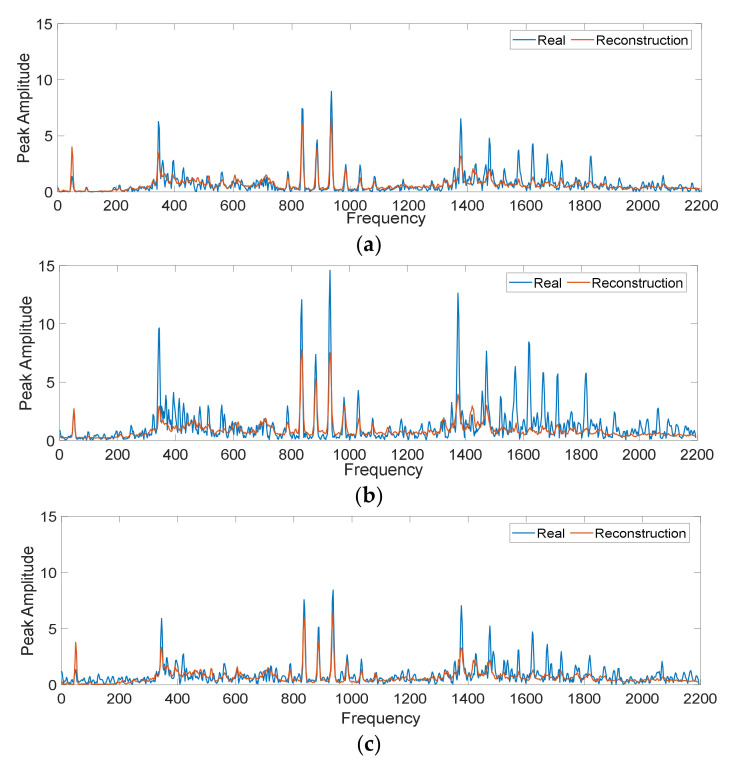
Characterization of vibration signals in F-D on operating conditions Ps2 and Lc2 for: (**a**) healthy condition, (**b**) healthy condition with root mean square (RMS) added (**c**) healthy condition with white Gaussian noise added.

Therefore, it is verified that the DNN-SAEs-based model is effectively characterizing the input signals correctly. Nevertheless, there is a concern in studies addressing techniques based on deep learning, and it is often considered “black box” approaches. Therefore, the high performance obtained through these approaches could be the result of unsuitable learning. In this regard, an intuitive study is carried out in which two parameters are modified in the acquisition signals. First, a relevance parameter, which it is expected to learn and it is adequately characterized, which is the root mean square (RMS). A common time domain fault detection technique for basic motor systems is to calculate the RMS values of the acquisition signals [47]. In this technique based on RMS value, when the RMS residual value exceeds a predefined threshold, a fault indication signal is generated. Therefore, it is intuited that a variation of this parameter is related to the state of the machine and it is important that a diagnostic approach has the ability to characterize the variations of this indicator. Second, there are parameters in the system that are undesirable to be characterized since they do not provide descriptive information for the diagnosis. In this regard, noise is one of these unwanted parameters, and it is desirable that a diagnostic approach does not characterize the noise variations. Therefore, the experiment consists of inducing variations of RMS and noise to the signals in time. To carry out the variations of the RMS value, first the ranges in which this parameter varies in our case studies are obtained. For the current signals, the maximum RMS value is 1.83 and the minimum value is 1.43. The mean RMS value for the healthy condition is 1.71, 1.46 is the average value for the bearing fault, 1.77 for the demagnetization fault, 1.48 the mean value for eccentricity fault, and 1.69 is the mean RMS value measured for gear fault. For the RMS value in vibration, 0.28 was obtained as maximum value and 0.13 as minimum value of RMS, with 0.14 being the average value for healthy condition, 0.25 the average value for bearing fault, 0.23 the average value for demagnetization fault, 0.15 the mean value for eccentricity fault and 0.16 is the mean RMS value measured for gear fault. In contrast, white Gaussian noise was added to the time signal vector in SNR of 10 to 100 db. An example of the characterization of a vibration signal in F-D in healthy conditions, in healthy conditions with the increased RMS and in healthy conditions with additional noise are shown in Figure 10.

From Figure 10b, it can be seen that increasing the RMS value causes an increase in the amplitude of the harmonics for the frequency spectrum, while in Figure 10c, it can be seen that the effect of increasing noise is only perceived in amplitude increase at low frequency values. For this practical case study, an MSE of 0.0097 was obtained for the reconstruction of the healthy condition, an MSE of 0.0120 for the reconstruction of the healthy condition with the modified RMS and an MSE of 0.0110 for the reconstruction of the healthy condition with noise added. These MSE values were similar for each of the test cases of the experiment. It should be noted that these MSE values are within the range of values obtained during the training process.

**Figure 11:**
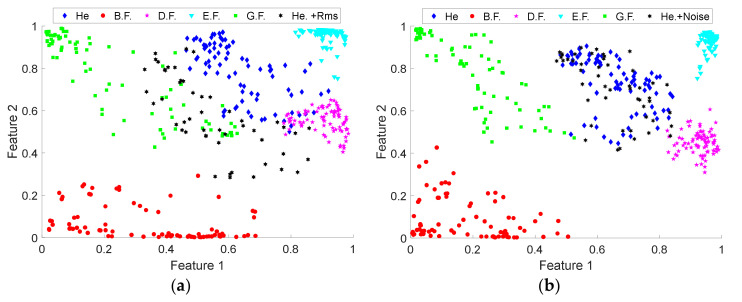
Projection of the extracted set of features resulting for: (**a**) each of the considered conditions and the healthy condition with a modified RMS (He. +RMS), (**b**) each of the considered conditions and the healthy condition with added noise (He. +Noise).

However, the repercussion of varying each of these parameters is perceived when performed to projections in the space of extracted features. In Figure 11, the space of features extracted through the DNN-based learning model is shown. The healthy condition and all the fault conditions considered for a case study are projected and additionally the features extracted from the healthy condition with an RMS modified are projected in Figure 11a. The features extracted from the healthy condition with white Gaussian noise added are projected in Figure 11b.

In the case of the healthy condition with an RMS modified (He +RMS), the RMS was increased to an average value of 0.16 in the time signal, while, for the healthy condition with noise added (He +Noise), white Gaussian noise was added with 50 dB SNR. When projecting the He+RMS features extracted from the learning model, it has a different dispersion to the healthy condition and is more similar to the condition whose RMS value is similar, only 3% are classified as healthy. This effect is desirable since it is expected that the diagnostic model will take into account the changes in the RMS value since the variation of this parameter is related to the state of the machine. In comparison, when are projecting the He+Noise features extracted from the learning model, they closely coincide with the healthy condition, obtaining 97% of classification as healthy samples, which shows that our diagnostic model does not characterize unwanted parameters, such as noise.

### 5.2. Comparison with Other Methods

Without performing a manual feature extraction, this study aims to diagnose multi-faults of an electromechanical system operating at different operating conditions using current and vibration data. A comparison between the proposed hybrid MDF by the DNN-SAEs-based model and classical machine learning approaches based on the extraction of features engineering (FE) and a DL-based technique is carried out. Diagnostic approaches based on FE offer simplicity of configuration and at the same time considerable diagnostic performance.

The establishment of the hyper-parameters of the ML approaches is simple and their application in electromechanical systems of low complexity is acceptable; however, the diagnostic accuracy of these approaches decreases in situations of high complexity. On the other hand, some DL-based techniques offer high classification accuracy; however, unlike the proposed method, the interpretation of the learning process in most of these methods is not clear, which comes to consider these approaches as “black boxes”.

In the case of classic ML approaches, the following stages are present: feature extraction, feature reduction, classification. A total of 15 statistical features in time domain, such as mean, maximum value, RMS, square root mean, standard deviation, variance, RMS shape factor, square root mean shape factor, crest factor, latitude factor, impulse factor, skewness, kurtosis, and normalized fifth and sixth. More details about the 15 TD statistical features and 3 FD features can be seen in [26,48], respectively. There are three statistical features in frequency domain, namely frequency center, RMS frequency and root variance frequency. Moreover, 19 fault frequency components, such as bearing faults frequencies, inner race defect frequency, outer race defect frequency, ball defect frequency, and gearbox faults frequencies, such as mesh frequency, mesh-related frequency are calculated. In this regard, the consideration of statistical time-domain and statistical frequency-domain features represents one of the most effective methods to provide fundamental information and a performance alternative to globally characterize the acquired signals. While the extraction of specific fault frequency component approaches offer greater sensitivity to fault occurrences. The feature reduction stage is carried out by different techniques, from which it can be distinguished whether the learning model is supervised or unsupervised. Moreover, if the resulting transformation is linear or non-linear. The most widely implemented methods include: linear discriminant analysis (LDA), which is supervised and linear, principal component analysis (PCA), the learning process and transformation of which is unsupervised and linear, respectively. In the case of non-linear and unsupervised methods, are Isomap and Laplacian Eigenmaps. Additionally, the classifiers used are a common artificial neural network (ANN) with a single hidden layer and the multi-class support vector machine (SVM). Moreover, recurrent neural networks (RNNs) are the deepest of all NNs, which can generate and address memories of arbitrary-length sequences of input patterns. RNN is able to build connections between units from a directed cycle. Due to the vanishing gradient problem during backpropagation for model training, LSTM is presented to prevent backpropagated errors from vanishing. Some parameters related to the methods for comparison are described below: (1) Isomap: the number of neighbors used for the geodesic distance computations is 12. (2) Laplacian Eigenmaps: the number of neighbors in the graph spectral is 10. (3) ANN: the number of neurons in the hidden layer set to 10. (4) SVM: an RBF kernel is applied. (5) LSTM: the number of the hidden units set to 300, Adam is used as an optimization method and the softmax layer is connected in the output layer as a classifier. For each one of the considered methods, 200 samples for each health condition are used. This database is divided into 70% for training, 20% for validation and 10% for test. The parameters were obtained through experimentation. The simulation environment for all algorithms performed in the experiments is as follows: MATLAB R2019a software environment.

Table 4 summarizes the average accuracies of feature engineering-based approaches in comparison with the proposed method under the same operating conditions and same fault states. The average accuracy of the proposed method is 92.03%. Specifically, the average accuracies of ANN and SVM based on FE combined with LDA are 85.61% and 86.26%, respectively, lower than that of the proposed method. In the same way, the results obtained through PCA with an ANN classifier (81.86%) and SVM (83.18%) are lower than the proposed method. As can be seen, the classifiers, ANN and SVM, have similar results for the same approaches, SVM is slightly higher than ANN, 0.61% and 1.32% for LDA and PCA, respectively. In the case of non-linear reduction techniques, Isomap and Lapacian were combined with the SVM and ANN classifier, respectively, obtaining average accuracies of 83.11% and 81.50%, correspondingly. The results are similar to those obtained by the linear LDA and PCA techniques. Compared with the proposed method, this is 8.92% higher than Isomap and 10.53% than Lapacian.

Compared to another DL method, LSTM performs better than classic ML methodologies. Regarding the proposed MDF fusion approach, LSTM is approximate but on average it is lower. Being the most approximate result for the operating conditions of Ps1 and Lc1 with a difference of just 0.3% between LSTM and the proposed approach. The greatest difference occurs for the operating condition of Ps2 and Lc2 with a difference of up to 5.8% of the proposed method above LSTM.

It can be concluded that: (1) the performance of the classical approaches based on machine learning depends heavily on the feature extraction stage. Furthermore, the different stages (extraction, reduction and classification) cannot be optimized at the same time. (2) The proposed method shows greater diagnostic precision than the other methods. The reason for this is that the proposed approach can adaptively learn the essential features of data from different sensors and also from different processing domains.

## 6. Conclusions

This paper presents a methodological process for the implementation of a diagnostic model for electromechanical systems based on the extraction and fusion of multiple sources of information. There are three important aspects to this methodology. The first is the learning of multi-sensor information features. The functionality of using multiple sources of information allows to better characterize the modes and different fault conditions and to better capture the variability of system operation. The second is the generation and optimization of a model based on deep learning techniques for the automatic coding and extraction of features. A GA optimizer is implemented to obtain hyper-parameters of a multiple SAE architecture in order to obtain the best possible characterization of the considered signals. The third is the ability to adapt to extract information from the most descriptive physical magnitude. Although in the methodological process a diagnosis methodology is developed and validated to identify and detect the occurrence of faults in a permanent magnet synchronous motor, the application of this proposed method is not limited to PMSM; even this method may be also applied to the monitoring of induction motors due to a high-performance characterization of the available signals being performed, and the methodology allows for an easy application and high adaptability to the available data. Based on all experimental studies, the effectiveness of the developed approach under different operating conditions and different healthy conditions is demonstrated. Additionally, is shown that the proposed method adaptively learns complex relationships from the acquired data from different sensor to characterize the different faults states. The results confirm that the proposed method was more effective than the existing methods.

The DNN SAE-based model learns multiple domain features that can be applied to analyze any type of signal derived from the electromechanical systems monitoring process. However, as a data-driven approach, the limitations of the present studies include the difficulty of configuring complex deep learning-based architectures to work properly. In this sense, the main contribution of the present methodology is an approach to be able to implement these techniques in a practical way in the study of electromechanical systems. With the rapid development of hardware technology, deep learning can be gradually applied in the new industrial framework. Future work will focus on using the learning functionality of this method to generate models that have the ability to incorporate new fault scenarios into the diagnostic process.

## Figures and Tables

**Table 1:** Parameter used in a stacked-SAEs-based DNN for vibration signals.

Feature Domain	Hyper-Parameters				
	L2 Regularization	Sparsity Regularization	Sparsity Proportion	First Hidden Layer	Second Hidden Layer
Time-Domain	5.0 × 10^−5^	5 × 10^−5^	0.4	670	208
Frequency-Domain	5.0 × 10^−6^	5 × 10^−3^	0.5	500	110
Time-Frequency-Domain	1.0 × 10^−5^	5.0 × 10^−5^	0.4	635	145

**Table 2:** Parameter used in Stacked-SAEs-based DNN for Current Signals.

Feature Domain	Hyper-Parameters				
	L2 Regularization	Sparsity Regularization	Sparsity Proportion	First Hidden Layer	Second Hidden Layer
Time-Domain	5.0 × 10^−6^	5.0 × 10^−6^	0.09	700	220
Frequency-Domain	5.0 × 10^−6^	5 × 10^−3^	0.05	600	150
Time-Frequency-Domain	5.0 × 10^−5^	5.0 × 10^−5^	0.1	750	160

**Table 3:** Resulting diagnostic accuracies.

Index	Index	Current				Vibration				Proposed Approach			
		TD	FD	TFD	Fusion	TD	FD	TFD	Fusion	TD	FD	TFD	Fusion
1	Ps1-Lc1	25.4	32.2	39.3	42.5	70.3	99.8	63.8	95.3	65.1	96.0	54.9	95.3
2	Ps1-Lc2	26.6	36.9	35.0	42.0	66.2	83.2	54.8	83.3	43.8	84.8	58.5	86.3
3	Ps2-Lc1	27.0	38.3	23.0	41.2	68.5	99.8	74.6	98.1	64.2	97.3	63.0	92.3
4	Ps2-Lc2	21.9	40.4	23.4	22.6	66.0	96.7	86.0	99.5	53.0	99.5	87.3	99.0
5	2Ps-Lc1	26.3	35.6	36.2	39.8	65.2	92.0	58.9	89.6	59.8	87.6	51.2	93.5
6	2Ps-Lc2	23.2	33.6	33.6	38.7	64.8	91.5	52.1	80.2	44.3	87.2	55.8	92.6
7	Ps1-2Lc	25.5	38.1	24.8	37.9	66.3	92.0	71.0	87.9	57.8	88.1	59.8	92.8
8	Ps2-2Lc	22.8	39.3	22.4	27.8	63.5	90.5	83.5	86.3	45.6	81.5	63.0	91.2
9	2Ps-2Lc	21.3	32.1	21.0	22.6	63.5	85.1	63.0	77.8	43.1	78.4	50.1	85.3
Average		24.4	36.2	28.7	35.01	66.03	92.2	67.5	88.6	52.9	88.9	60.4	92.03

**Table 4:** Diagnosis performances achieved by compared methods in a fault diagnosis task.

Index	Index	Methods in Fault Diagnosis							
		LDA + ANN	LDA + SVM	PCA + ANN	PCA + SVM	Isomap + SVM	Laplacian + ANN	LSTM	Proposed Method
1	Ps1-Lc1	92.5	93.1	90.5	91.3	98.0	93.0	95.0	95.3
2	Ps1-Lc2	88.6	81.9	76.7	77.8	86.3	85.6	85.1	86.3
3	Ps2-Lc1	91.3	91.1	88.1	89.3	84.6	86.0	89.2	92.3
4	Ps2-Lc2	88.5	85.9	90.5	89.6	87.9	85.0	93.2	99.0
5	2Ps-Lc1	83.5	86.0	81.2	82.6	78.6	75.6	90.5	93.5
6	2Ps-Lc2	76.5	84.3	77.0	81.1	79.6	78.3	92.0	92.6
7	Ps1-2Lc	85.5	85.0	80.3	82.6	78.5	79.6	90.0	92.8
8	Ps2-2Lc	88.4	89.8	81.5	816	77.5	78.9	88.0	91.2
9	2Ps-2Lc	75.7	79.3	71.0	73.1	77.0	71.5	80.1	85.3
Average		85.61	86.26	81.86	83.18	83.11	81.50	88.63	92.03
